# Supplementary material for: RAAS-deficient organoids indicate delayed angiogenesis as a possible cause for autosomal recessive renal tubular dysgenesis
Source: Nat Commun. 2023 Dec 9;14:8159. doi: 10.1038/s41467-023-43795-x (PMC10710424; doi:10.1038/s41467-023-43795-x)
Supplement: Supplementary file 1 — Supplementary Information file [file 41467_2023_43795_MOESM1_ESM.pdf]

RAAS-deficient Organoids Indicate Delayed Angiogenesis as a possible cause for Autosomal Recessive Renal Tubular Dysgenesis

Naomi Pode-Shaked[1,2,3,4], Megan Slack[1,2], Nambirajan Sundaram[5], Ruth Schreiber[6], Kyle Mc'Cracken[1,2], Benjamin Dekel[4,7], Michael Helmrath[5], Raphael Kopan[1,2,*]

[1]Department of Pediatrics, University of Cincinnati College of Medicine, Cincinnati, OH, 45229, USA

[2]Division of Developmental Biology, Cincinnati Children's Hospital Medical Center, Cincinnati, OH, 45229, USA

[3]Division of Nephrology and Hypertension, Cincinnati Children's Hospital Medical Center, Cincinnati, Ohio, USA.

[4]Sackler Faculty of Medicine, Tel Aviv University, Israel

[5]Division of Pediatric Surgery, Cincinnati Children's Hospital Medical Center, Cincinnati, OH, 45229, USA

[6]Department of Pediatrics, Soroka University Medical Center, Ben Gurion University of the Negev, Beer Sheva, Israel.

[7]Pediatric Stem Cell Research Institute and division of pediatric nephrology, Edmond and Lily Safra Children's Hospital, Sheba Medical Center, Ramat-Gan, Israel.

*Author for correspondence: Rafi.kopan@gmail.com

Supplemental information – Table of content:

SUPPLEMENTAL FIGURES AND LEGENDS 2

FIGURE S1| 3

FIGURE S2| 4

FIGURE S3| 5

FIGURE S4| 7

FIGURE S5| 8

FIGURE S6| 9

SUPPLEMENTAL TABLES 11

TABLE S1: ANTIBODIES USED IN THIS MANUSCRIPT..11

TABLE S2: PRIMERS AND GUIDE RNAS (GRNA) USED IN THIS MANUSCRIPT..13

TABLE S3: NUMBER OF MICE USED FOR ORGANOID TRANSPLANTATIONS..15

SUPPLEMENTAL NOTE 16

AR-RTD patient's phenotype description..16

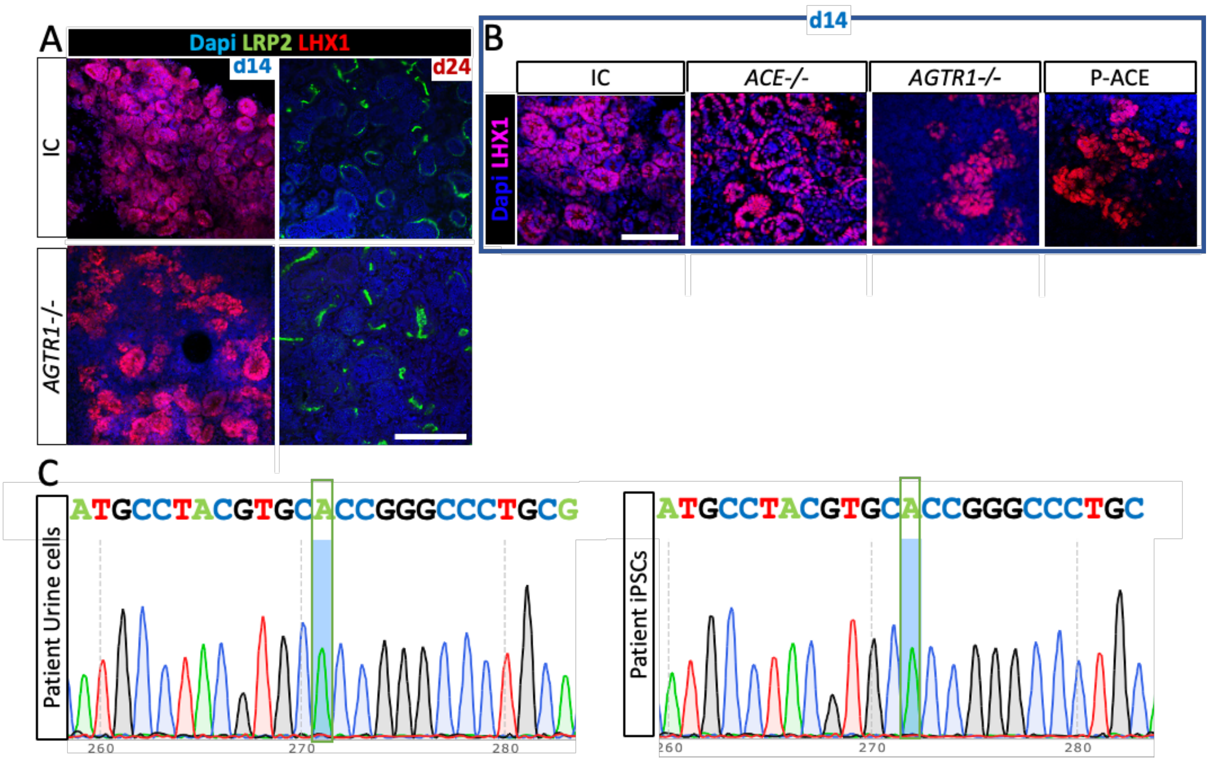

Fig. S1, supporting Figures 1 and 2. (A) Representative IF staining for the renal vesicle marker, LHX1 and the proximal tubule marker, LRP2 in isogenic control (IC) and *AGTR1*-/- d14 and d24 organoids. 100µm Scale bar for all the images in (A) is placed in the *AGTR1-/-,* d24 panel. (B) Representative IF staining of IC, *ACE*-/-, *AGTR1*-/- and P-ACE organoids at day 14 of the differentiation protocol for LHX1 (red). 100µm Scale bar for all the images in (B) is placed in the IC panel. (C) Sequencing DNA from donor urine cells and reprogrammed derivatives contain the biallelic c.2570G>A missense mutation in the *ACE* gene.

Abbreviations: P-ACE- iPSC derived from urine cells of a patient with biallelic pathogenic variant in the *ACE* gene. C-ACE- iPSC in which the c.2570G>A *ACE* variant has been corrected with CRISPR/Cas9.

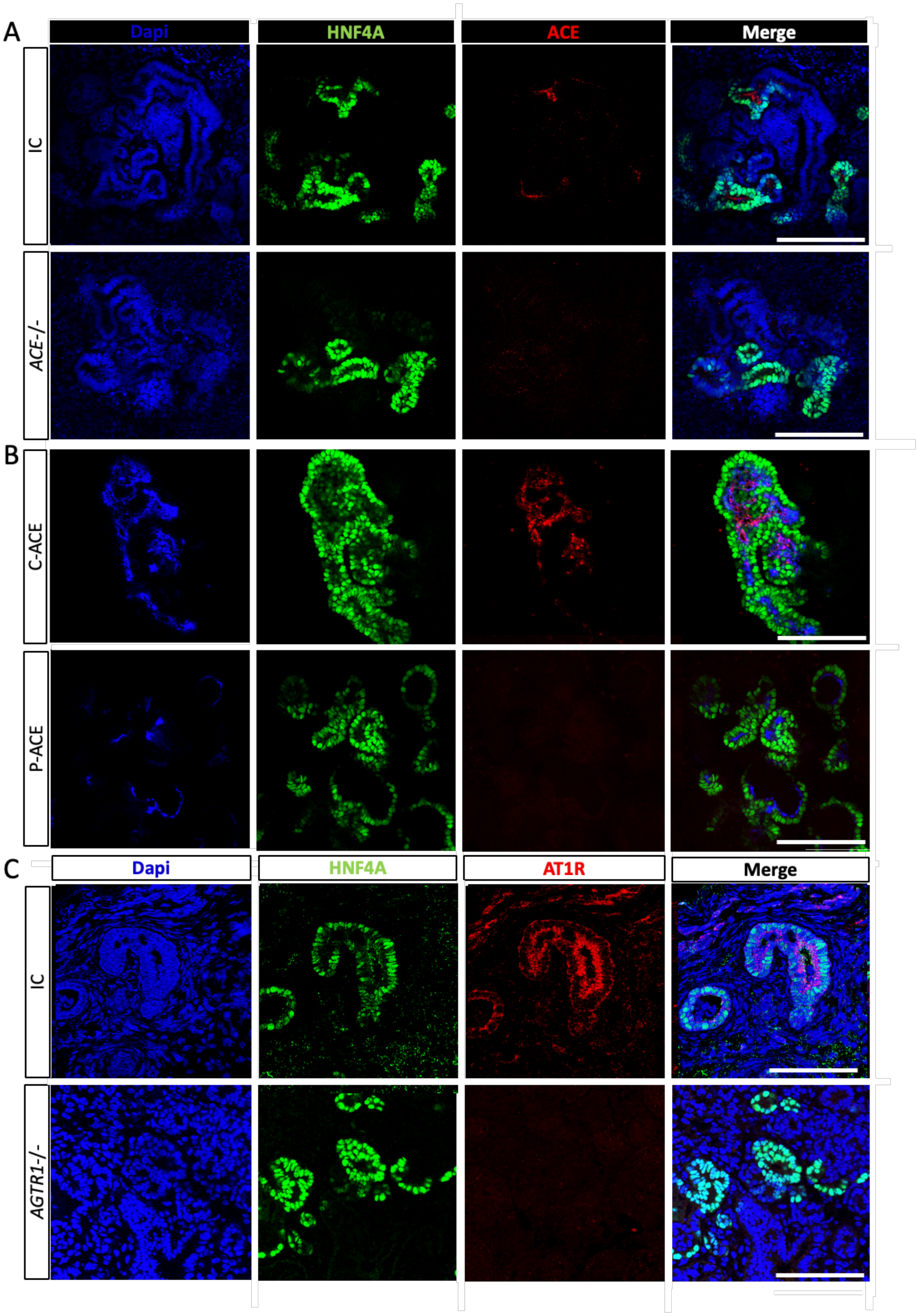

Fig. S2, supporting Figure 2. Detection of ACE and AT1R in iPSC-derived organoids from mutant lines and Isogenic Controls (IC). (A-B) Immunofluorescence staining for ACE in *ACE*-/- and P-ACE iPSC-derived organoids compared with their corresponding ICs (IC, C-ACE) for ACE. (C) Staining for AT1R in *AGTR1*-/- and IC iPSC-derived organoids. IC=Isogenic control. P-ACE=AR-RTD patient-derived iPSC. C-ACE=CRISPR-corrected iPSCs. Scale bars=100µm are shown in the merged images (right).

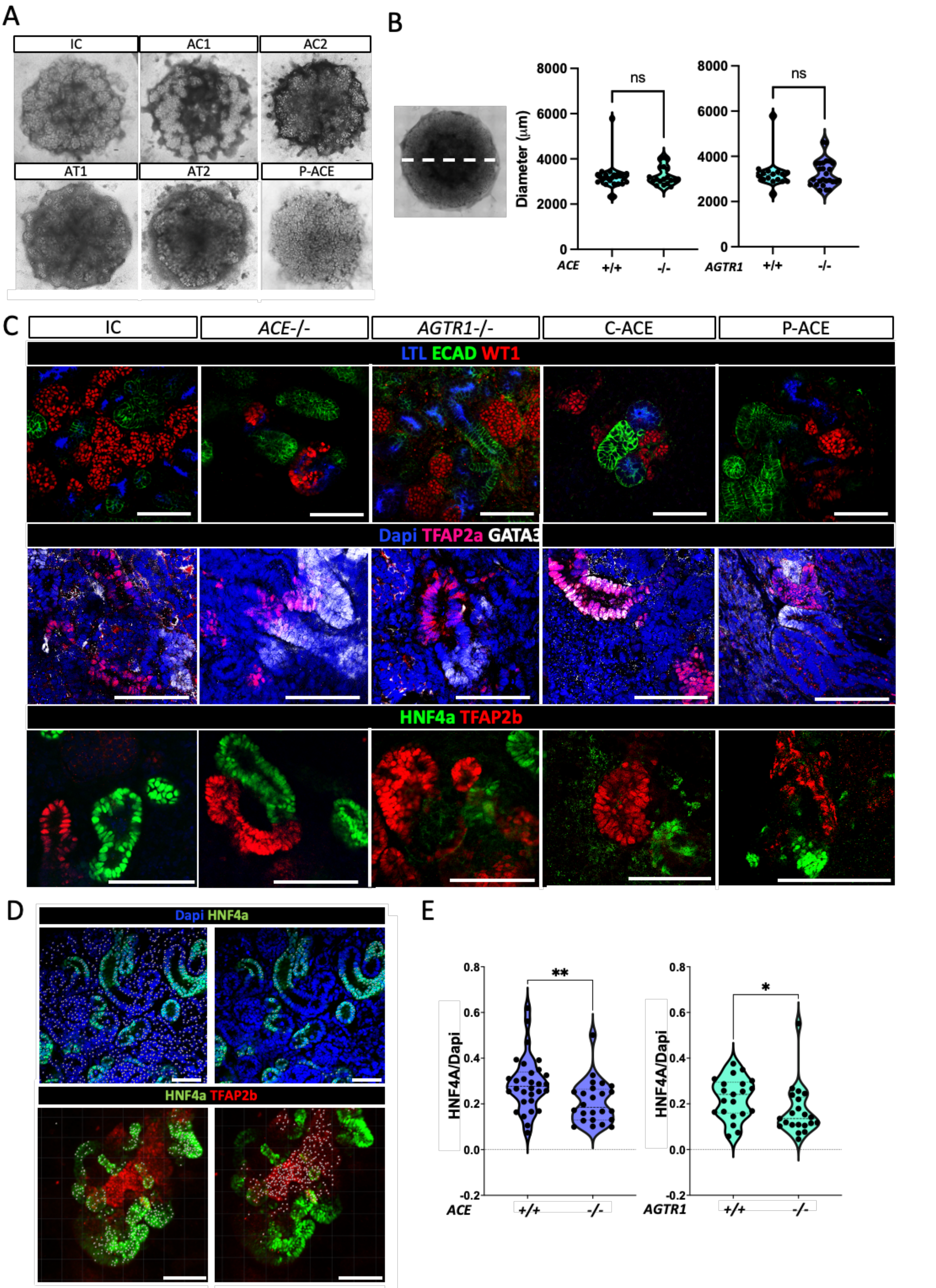

Fig. S3, supporting Figure 3. Characterization of *ACE-/-*, *AGTR1-/-* and P-ACE iPSC derived organoids. (A) Representative bright field images of Isogenic Control (IC), *ACE -/-*, *AGTR -/-,* and P-ACE iPSC-derived organoids. (B) Diameter distribution calculated for *ACE-/-* and *AGTR1-/-* organoids relative to their respective ICs. Calculations were performed on

n=19 *ACE*-/-, n=26 IC (*ACE*+/+), n=26 *AGTR1*-/- and n=16 IC (*AGTR1*+/+) organoids from n=4 biologically independent differentiation experiments. Graphs present the mean ±S.E.M of organoid diameters; Comparisons were performed using a two-sided t-test. ns=not significant (*p-value>0.05)*. (C) Immunofluorescence staining of *ACE*-/-, *AGTR1*-/- and patient (P-ACE) iPSC-derived organoids compared with their corresponding ICs (IC, C-ACE) for PT (LTL, HNF4a), LOH/DT/CT (TFAP2a/b, ECAD, GATA3) and podocytes (WT1). (D) Representative section from a z-stack of HNF4a, TFAP2b, and Dapi stain IC organoids analyzed with IMARIS. Shown are individual cells identified by the software for HNF4a+ and Dapi+ quantification (top) or HNF4a+ and TFAP2b+ (bottom). (E) Graphs display the mean ratio of HNF4a (PT cells) to Dapi positive cells (all cells) in *ACE*-/- or *AGTR1*-/- organoids and their respective ICs. Each dot represents the mean HNF4a+/Dapi+ ratio of x4 z-sections per organoid. Quantification was performed on n=21 organoids for *ACE*-/- or IC and on n=24 organoids for *AGTR1*-/- or n=30 for respective IC from n=4 biologically independent differentiation experiments. Data is presented as mean ±S.E.M of the HNF4a+/Dapi+ ratio; Comparisons were performed using a two-sided t-test. **p*=0.013, ***p*=0.004. Scale bars=100μm are indicated in each image. IC=Isogenic Control. PT- Proximal Tubules, DT- Distal Tubules, LOH- Loop of Henle, LTL- Lotus tetragonolobus lectin, HNF4a - Hepatocyte Nuclear Factor 4α, TFAP2b - Transcription factor AP-2 beta. Source data for Figures S3B and E are provided as a Source Data file.

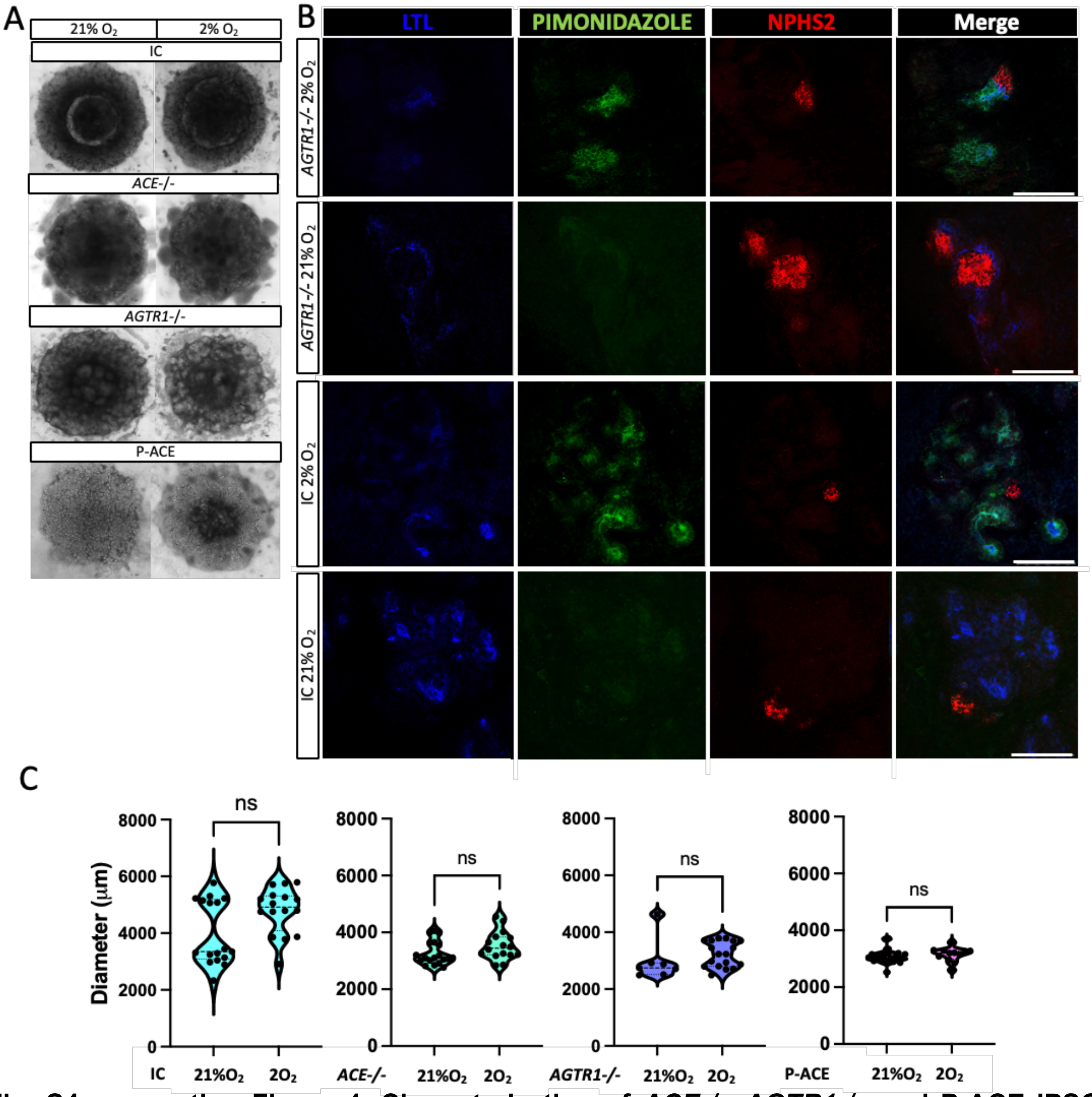

Fig. S4, supporting Figure 4. Characterization of *ACE-/-*, *AGTR1-/-,* and P-ACE iPSC derived organoids grown in standard versus hypoxic conditions. (A) Representative bright field images of IC, *ACE -/-*, *AGTR -/-,* and P-ACE iPSC-derived organoids grown in 21%O_2 or hypoxic (2%O_2) conditions. (B) Immunofluorescent staining for pimonidazole (hypoxia marker, green), LTL (blue) and NPHS2 (red), in *AGTR1*-/- and IC organoids grown in standard (21%O_2) or hypoxic (2%O_2) conditions. Scale bars=100μm are indicated in each image. (C) Diameter of IC, *ACE-/-*, *AGTR1-/-,* and P-ACE iPSC-derived organoids grown in 21% O_2 compared to 2% O_2. Calculations were performed on IC organoids grown in 21% O_2 (n=20) or 2% O_2 (n=19), *ACE*-/- organoids grown in 21% O_2 (n=19) or 2% O_2 (n=16), *AGTR1*-/- organoids grown in 21% O_2 (n=7) or 2% O_2 (n=18) and P-ACE organoids grown in 21% O_2 (n=26) or 2% O_2 (n=12) from n=4 biologically independent differentiation experiments. Graphs present the mean ±S.E.M of organoid diameters. Comparisons were performed using a two-sided t-test. ns=not significant (*p-value>0.05)*. Scale bars=100μm are indicated in each merged image. P-ACE=AR-RTD patient-derived iPSC. Source data for Figure S4C are provided as a Source Data file.

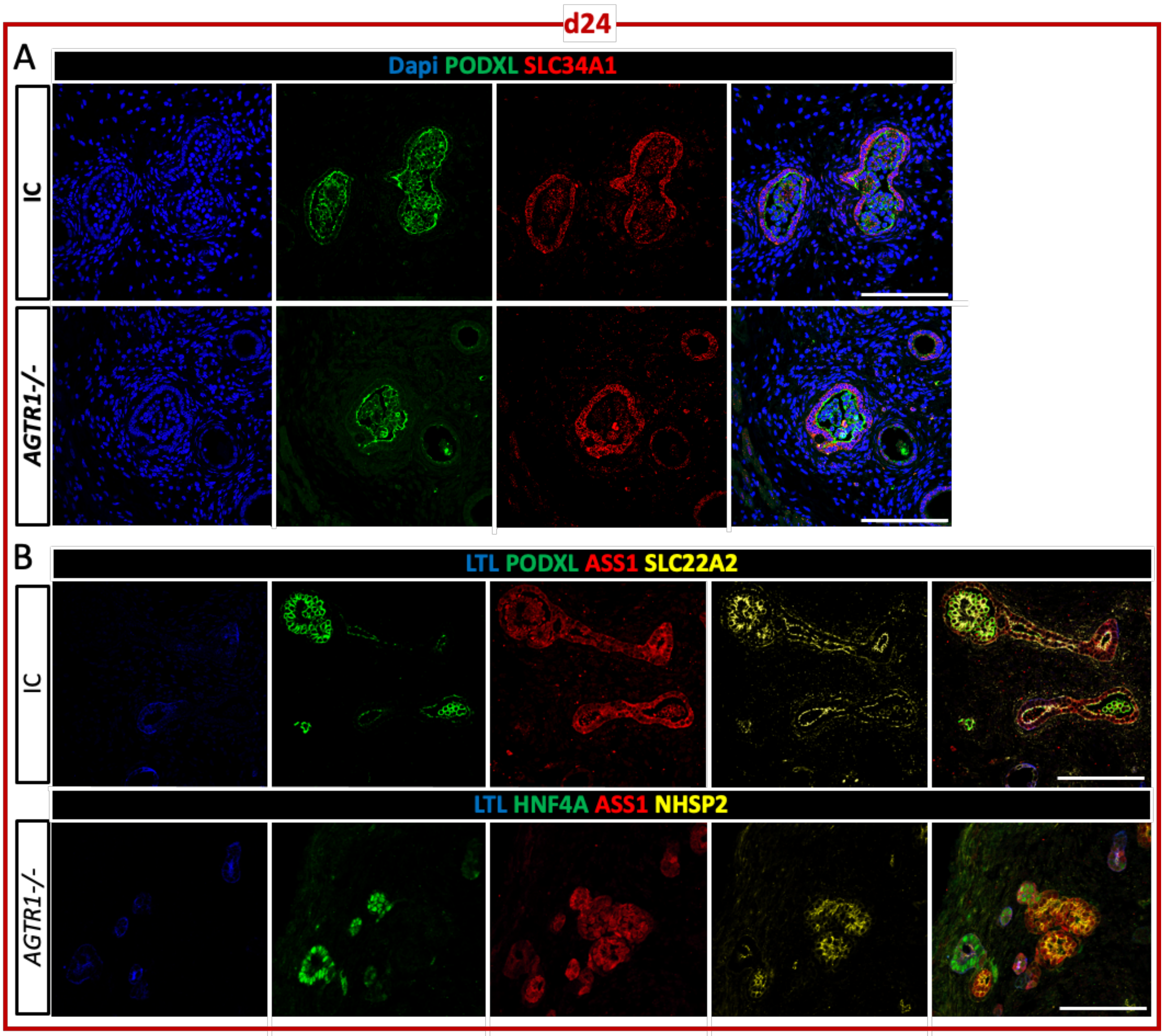

Fig. S5, supporting Figure 5. Characterization of *AGTR1-/-* and IC iPSC-derived organoids transplanted at d24 under the kidney capsule of immunodeficient mice. (A) IF staining of *AGTR1-/-* and IC extracted organoids for Dapi, PODXL (podocyte) and SLC34A1 (PT). (B) IF staining of *AGTR1-/-* and IC extracted organoids for LTL, PODXL, ASS1, SLC22A2, HNF4a and NPHS2. Scale bars=100µm are indicated in the images.

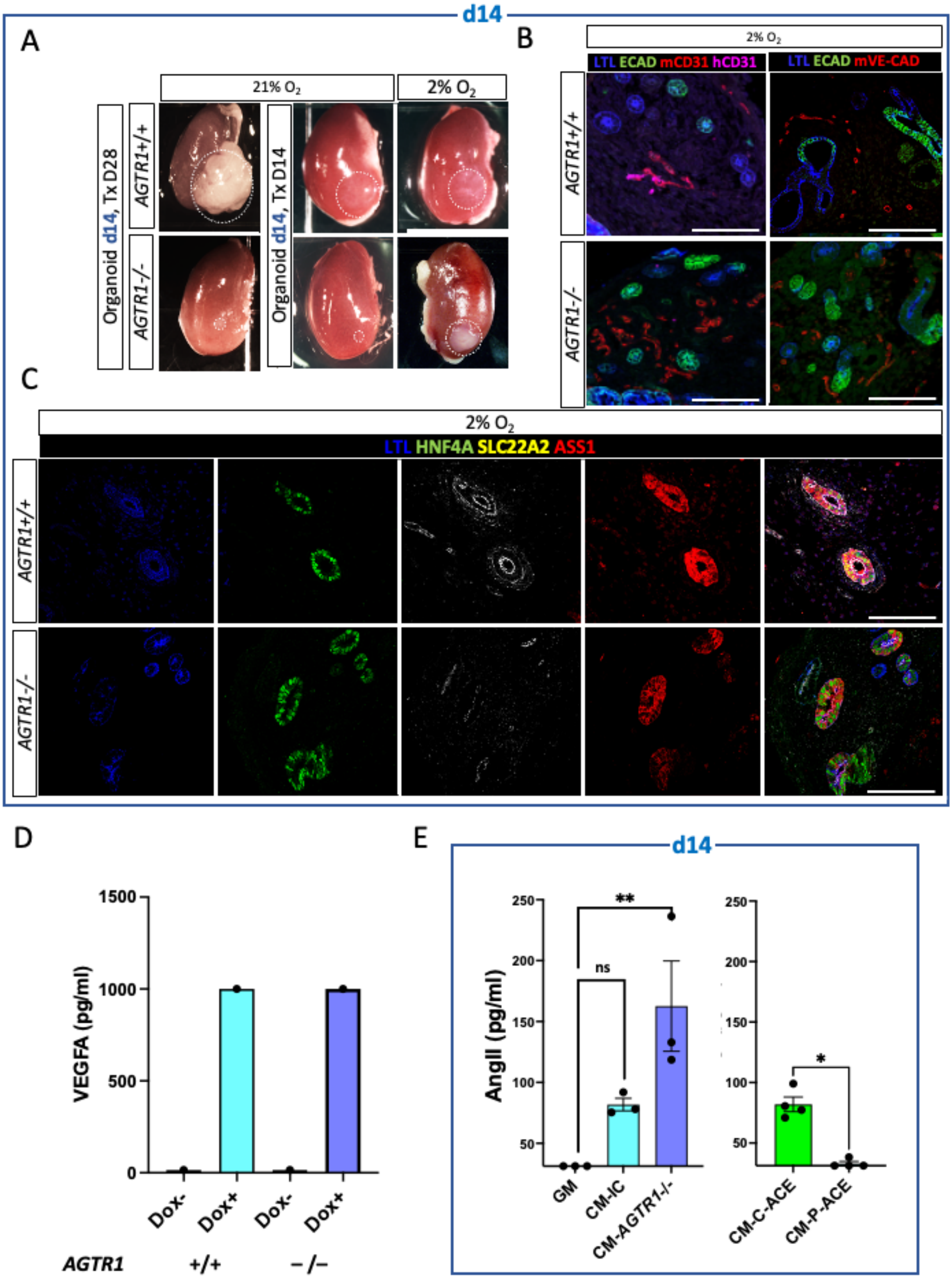

Fig. S6, supporting Figures 6 and 7. (A) Images of Isogenic Control (IC) and *AGTR1*-/- iPSC derived organoids grown at 21%O_2 or 2%O_2, transplanted at d14, and extracted at d28 (TxD28) or d14 (TxD14). (B) IF staining of explanted *AGTR1*-/- and IC-derived organoids (grown in 2%O_2 prior to transplantation at day 14) for mouse CD31 (mCD31), human CD31 (hCD31) or mVE-Cadherin (mVE-CAD). (C) IF staining for Proximal Tubule (PT) maturation

markers (LTL, HNF4a, SLC22A2 and ASS1) in explanted *AGTR1*-/- and IC-derived organoids, grown in 2%O_2 prior to transplantation at d14. Scale bars=100μm are indicated in the merged images. (D) ELISA assay for detection of human VEGF-A protein in conditioned media (CM) from *AGTR1*-/- and IC (+/+) iPSCs infected with lentivirus harboring a doxycycline (Dox)-inducible hVEGF-A construct and selected with Blasticidin. Cells were either treated with Dox for 48h (Dox+) or left untreated (Dox-). Results are presented as the mean ±S.E.M of n=3 biologically independent experiments. In all experiments, VEGF-A was undetectable without Dox induction (<15.6pg/ml) and expressed over the upper limit detected by the assay (1000pg/ml) following Dox induction. (E) Quantification of Angiotensin II (AngII) protein secretion in growth media (GM) or in CM from day 14 IC (CM-IC), *AGTR1*-/- (CM-AGTR1-/-), C-ACE (CM-C-ACE) or P-ACE (CM-P-ACE) organoids. Results are presented as the mean±S.E.M of n=3 biologically independent experiments for GM, CM-IC, and CM-*AGTR1*-/- and n=4 biologically independent experiments for C-ACE and P-ACE. Comparisons between GM, CM-IC, and CM-*AGTR1*-/- were performed using one-way ANOVA on ranks. P-values were adjusted for multiple comparisons using the two-stage step-up method of Benjamini, Krieger and Yekutieli. ns=non-significant (*p*=0.09). ***p*=0.007. Comparison between C-ACE and P-ACE was performed using a two-sided t-test. **p*=0.03. The lower limit of detection of AngII by the ELISA kit was 31.25pg/ml. P-ACE=AR-RTD patient-derived iPSC. C-ACE=CRISPR-corrected iPSCs. Source data for Figure S6D and E are provided as a Source Data file.

Supplementary Table 1: Antibodies used in this manuscript.

Antibody	Source	Catalog/clone Number	Dilution factor
**Immunofluorescence**			
Anti Human ACE	Sigma-Aldrich	HPA029298	1:200
Anti Human AGTR1	Bioss Antibodies	BS-2097R	1:200
Anti Human ASS1	Abcam	ab77590	1:100
Anti Human CD31	Novus Biologicals	NBP2-80640	1:200
Anti Human CUBN	Sigma-Aldrich	SAB4301904	1:100
Anti Human E-Cadherin	BD Transduction Laboratories™	610182	1:350
Anti Human GATA3	BioTechne R&D	AF2605	1:500
Anti Human HNF4a	Abcam	ab41898	1:200
Anti Human Kim1	R&D Systems	AF1750	1:200
Anti Human KRT8/18	Abcam	ab194130	1:1000
Anti Human LIM1/LHX1	Abcam	ab229474	1:250
Anti Human LRP2/Megalin	BioTechne R&D	MAB9578-100	1:100
LTL (Biotinylated)	Vector Laboratories	B-1325-2	1:500
Anti Human Nephrin/NPHS1	R&D Systems	AF4269	1:300
Anti Human Podocalyxin	BioTechne R&D	MAB1658	1:50
Anti Human Podocin/NPHS2	Proteintech	20384-1-AP	1:100
Anti Human SLC22A2	Abcam	ab170871	1:100
Anti Human SLC34A1	ThermoFisher	PA5-62358	1:100
Anti Human TFAP2B	Cell Signaling Technology	2509S	1:200
Anti Human TROMA-1	Sigma-Aldrich	MABT329	1:50
Anti Human VE-Cadherin	Novus Biologicals	NBP1-43347	1:200
Anti Human VEGF-A	Abcam	ab52917	1:250
Anti Human WT1	Santa Cruz	sc-393498	1:20
Anti mosue VE Cadherin	Novus Biologicals	AF1002-SP	1:200
Anti mosue CD31	Cell Signaling Technology	77699T	1:200
**Seconadry antibodies**			

Donkey Anti-Rabbit IgG H&L (Alexa Fluor 488)	Abcam	ab150073	1:400
Donkey Anti-Mouse IgG H&L (Alexa Fluor 488)	Abcam	ab150105	1:400
Donkey Anti-Goat IgG H&L (Alexa Fluor 647)	Abcam	ab150131	1:400
Donkey Anti-Rat IgG H&L (Alexa Fluor 647)	Abcam	ab150155	1:400
Donkey Anti-Rabbit IgG H&L (Alexa Fluor 568)	Abcam	ab175470	1:400
Donkey F(ab')2 Anti-Mouse IgG H&L (Alexa Fluor 568)	Abcam	ab175699	1:400
Streptavidin, Alexa Fluor™ 405 conjugate	Invitrogen	S32351	1:400
**Flow Cytometry**			
Anti human ACE conjugated to APC	Miltenyi Biotech	130-108-014	1:11
human IgG1 Isotype control	Miltenyi Biotech	130-113-446	1:50
Anti human ATIR conjugated to APC	Novus Biologicals	FAB10244A	1:11
IgG2b kappa Isotype Control	eBioscience™	17-4031-82	1:100
7-AAD	eBioscience™	00-6993-50	1:11

Supplemental Table 2: Primers and guide RNAs (gRNA) used in this manuscript.

Gene	Forward	Reverse
VEGF-A	AGGGCAGAATCATCACGAAG	AGGGTCTCGATTGGATGGCA
VEGF-A165	GAGCAAGACAAGAAAATCCC	CCTCGGCTTGTCACATCTG
VEGF-A121	AGGCCAGCACATAGGAGAGA	GCCTCGGCTTGTCACATTTTT
VEGF-A189	TAAGTCCTGGAGCGTTCCCT	ACGCGAGTCTGTGTTTTTGC
ACE flanking the deletion (Exon 10 to intron 15)	GCCTGAAACTCCCTCTTCCAG	CCATTTGGAGTCCAAGCCCATG
ACE within the deletion (intron 13)	CTTGAAGCCAGGAGTTGGAG	CCTTAGGAAGGAGCCAGCTT
AGTR1 flanking the deletion	AGGCTTTATCAGTTCACAGTGT	TGTGGCTTTGCTTTGTCTTGT
AGTR1 within the deletion	ACTCACGTGTCTCAGCATTG	TCACGTATGATGCCTAGTTGAA
GAPDH	CAATGACCCCTTCATTGACC	GACAAGCTTCCCGTTCTCAG
hSLC22A2	AGGGTCTCGATTGGATGGCA	GTTAAACTCGGTGACGATGGAC
hSLC34A1	CCCTCAGGTCCTACACAGGAT	GGAGCAGACGAAGAGGTAGAG
hSLC22A5	TCCACCATTGTGACCGAGTG	ACCCACGAAGAACAAGGAGATT
hSLC5A12	AGGCAACTTCCCGAGAGTTC	CCCCAAAGCGGTAGACTTCAG
hSLC7A13	TTGGCTCCACGGTTGCTTTT	CCAATGCCAGACATTTCTTAGG C
ACE Exon 17 for c.2570 A>G sequencing	GCAGGTAATGTGGTGTTGGG	GGTGACTTGCCCGACATAGA
gRNA sg320	GTTTCGTTTCGGGTAACAGG AGG	
gRNA sg321	GCATGGTATGAAGTAGGTGC CGG	
gRNA Sg431	GAGAATCATTTTGATCACCTGGG	
gRNA Sg433	TTGGTAGTGAAGTGCTGCAGAGG	
gRNA containing the *ACE* c.2570 A>G mutation	CCTGCATGCCTACGTGCaCC	
Donor sequence used for correction of ACE c.2570 A>G mutation	C*C*C*GACATGTGCCCTTACCCAC AGGTGAGCAGGAATGGCCCCTC CAGGTTGATGTGCTGGGCCCCGT AGTGACGGTGCAGGGCgCG GCGCACGTAGGCATGCAGGTTGA GGTAGAGTGGCGACATGTGCCCT TACCCAGCAGGTGAGCAGGAATG GGCCCCTCCAGGTTGATGTGCTG GGCCCCGTAGTGACGGTGCAGG GCGCGGcGCACGTAGGCATGCAG GTTGAGGTAGAGTGGC*T*G*C.	

hACE_R857H_gen	5'-AGGTAATGTGGTGTTGGGAG-3'	5'-ACCCTCTAGTCAGCCCTGTC-3'
Primers for cloning of hVEGF-A	ggggACAAGTTTGTACAAAAAAGCAGGCTatgaactttctgctgtcttgggtgc	ggggACCACTTTGTACAAGAAAGCTGGGTtcaccgcctcggcttgtcac

Supplemental Table 3: Number of mice used for organoid transplantations.

No. of recipient mice for organoid transplantation, per growth condition (engrafted organoids/total no. implanted)			
**Oxygen:**	**21% O2**		**2% O2**
**iPSC line**	**Transplantation of d24 organoids**	**Transplantation of d14 organoids**	
**Isogenic Control (IC)**	**4 (4/4)**	**22 (22/22)**	**4 (4/4)**
AGTR1-/-	**4 (4/4)**	**22 (0/22)**	**6 (6/6)**
***AGTR1-/-* + Dox-inducible VEGF-A**	**–**	**4 (4/4)**	**–**

Supplemental Note:

AR-RTD patient's phenotype. The patient is a female, born at 30 weeks of gestation following a pregnancy complicated with oligohydramnios (AFI 2, N 8-18) and IUGR since 22nd weeks of gestation. Following birth, she was anuric and developed hyperkalemia for which peritoneal dialysis was initiated. She required Dopamine for profound hypotension and Fluconazole. ACE serum levels were undetectable, Renin serum levels were elevated >5000uIU/ml, Aldosterone was at the normal range. Following treatment with Fludrocortisone and blood pressure stabilization kidney function was partially recovered. She was discharged from the NICU at age 3 months and continues close follow-up by the pediatric nephrology unit at the Soroka Medical Center. She is currently 12 years old with CKD stage 3. ACE level remains undetectable and Renin remains >5000uIU/ml. Kidney ultrasound remains unremarkable apart for simple cysts (x1 in each kidney). IUGR- Intra-uterine growth restriction.
