# Supplementary material for: RAAS-deficient organoids indicate delayed angiogenesis as a possible cause for autosomal recessive renal tubular dysgenesis
Source: Nat Commun. 2023 Dec 9;14:8159. doi: 10.1038/s41467-023-43795-x (PMC10710424; doi:10.1038/s41467-023-43795-x)
Supplement: Supplementary file 12 — Reporting Summary [file 41467_2023_43795_MOESM12_ESM.pdf]

nature portfolio

Corresponding author(s): Raphael Kopan

Last updated by author(s): 10/24/2023

Reporting Summary

Nature Portfolio wishes to improve the reproducibility of the work that we publish. This form provides structure for consistency and transparency in reporting. For further information on Nature Portfolio policies, see our Editorial Policies and the Editorial Policy Checklist.

Statistics

For all statistical analyses, confirm that the following items are present in the figure legend, table legend, main text, or Methods section.

n/a	Confirmed	
☐	☒	The exact sample size (*n*) for each experimental group/condition, given as a discrete number and unit of measurement
☐	☒	A statement on whether measurements were taken from distinct samples or whether the same sample was measured repeatedly
☐	☒	The statistical test(s) used AND whether they are one- or two-sided<br>*Only common tests should be described solely by name; describe more complex techniques in the Methods section.*
☐	☒	A description of all covariates tested
☐	☒	A description of any assumptions or corrections, such as tests of normality and adjustment for multiple comparisons
☐	☒	A full description of the statistical parameters including central tendency (e.g. means) or other basic estimates (e.g. regression coefficient) AND variation (e.g. standard deviation) or associated estimates of uncertainty (e.g. confidence intervals)
☐	☒	For null hypothesis testing, the test statistic (e.g. *F*, *t*, *r*) with confidence intervals, effect sizes, degrees of freedom and *P* value noted<br>*Give P values as exact values whenever suitable.*
☒	☐	For Bayesian analysis, information on the choice of priors and Markov chain Monte Carlo settings
☒	☐	For hierarchical and complex designs, identification of the appropriate level for tests and full reporting of outcomes
☒	☐	Estimates of effect sizes (e.g. Cohen's *d*, Pearson's *r*), indicating how they were calculated

Our web collection on statistics for biologists contains articles on many of the points above.

Software and code

Policy information about availability of computer code

Data collection	The raw bulk RNA-seq data, consisting of paired-end FASTQ files, were processed prior to alignment using the command line tool fastp (version 0.23.2). kallisto (version 0.46.0) was used to pseudoalign the processed reads against the hg38 transcriptome and quantify transcript abundances. The R package tximport (version 1.22.0) is then used to estimate gene counts from the kallisto output, generating count/TPM.<br>- Z-section images of Immunofluorescent stained kidney organoids were captured via NIS-Elements software on a Nikon A1 inverted confocal microscope.
Data analysis	- RNA sequencing: Differentially Expressed Genes (DEG) between WT and mutants were found using the R package DESeq2 (version 1.38.3).<br>- Quantification of proximal tubule (PT), distal tubule (DT) and total cell number (Dapi) in kidney organoids was performed via Imaris visualization and analysis software (versions 9.9.1 and 10).<br>- T-test and ANOVA were performed in PRISM9 software (version 9.4.0).

For manuscripts utilizing custom algorithms or software that are central to the research but not yet described in published literature, software must be made available to editors and reviewers. We strongly encourage code deposition in a community repository (e.g. GitHub). See the Nature Portfolio guidelines for submitting code & software for further information.

Data

Policy information about availability of data

All manuscripts must include a data availability statement. This statement should provide the following information, where applicable:

- Accession codes, unique identifiers, or web links for publicly available datasets
- A description of any restrictions on data availability
- For clinical datasets or third party data, please ensure that the statement adheres to our policy

RNA sequencing data have been deposited in the National Center for Biotechnology Information Gene Expression Omnibus (GEO) under the GEO Series accession number GSE229842 [https://www.ncbi.nlm.nih.gov/geo/query/acc.cgi?&acc=GSE229842]. Supplementary Data (including Supplementary Note, table and Data) contain details of all essential resources used in this manuscript. All remaining data that supports the findings of this research is either comprehensively included in the manuscript itself, the Supplementary Information annexes, or in Source data provided with this paper.

Research involving human participants, their data, or biological material

Policy information about studies with human participants or human data. See also policy information about sex, gender (identity/presentation), and sexual orientation and race, ethnicity and racism.

Reporting on sex and gender	There is no indication that AR-RTD has a sex based bias. Our human iPSC lines are all female (XX) derived. Our mechanistic study is not designed to understand if gender-based differences exist.
Reporting on race, ethnicity, or other socially relevant groupings	A single AR-RTD patient donor was used.<br>Anonymized NHSK cells were collected from a healthy donor under CCHMC IRB protocol CR1 2008-1331 and reprogrammed at CCHMC to iPSCs in 2011.
Population characteristics	A single AR-RTD patient donor was used.
Recruitment	Urine derived renal epithelial cells were collected from a 9-year-old female donor with AR-RTD following signed informed consent from the patient's legal guardian
Ethics oversight	The studies involving human participants were reviewed and approved by the Sheba Medical Center, the Soroka Medical Center, and the Cincinnati Children's Hospital Medical Center Ethics Committees.

Note that full information on the approval of the study protocol must also be provided in the manuscript.

Field-specific reporting

Please select the one below that is the best fit for your research. If you are not sure, read the appropriate sections before making your selection.

☒ Life sciences ☐ Behavioural & social sciences ☐ Ecological, evolutionary & environmental sciences

For a reference copy of the document with all sections, see nature.com/documents/nr-reporting-summary-flat.pdf

Life sciences study design

All studies must disclose on these points even when the disclosure is negative.

Sample size	- We modeled 2 of the 4 AR-RTD causative genes (ACE and AGTR1)<br>- We used two genomes for ACE: the patient's and a CRISPR-deleted in human induced pluripotent stem cell line (NHSK). we have isogenic controls for all mutant lines.<br>- Each test was performed with multiple replicates as specified in the manuscript
Data exclusions	No data was excluded from the analyses.
Replication	- Each test was performed with multiple replicates as specified in the manuscript<br>- Transplantation were performed interdependently and in >3 replicates<br>- Multiple organoids were generated and characterized on different dates with different reagents batches as detailed in the text<br>- No outlier were identified or excluded
Randomization	The study of how different genotypes impacts PT development cannot be randomized. The number of replicates and the design account for the effect of covariants (i.e. date, batch, genome, line)
Blinding	- Quantification of proximal tubule/distal tubule cell ratio and proximal tubule/dapi+ cell ratio was perfomed by an observer blinded to

Blinding

organoid genotype identity.
- Organoid transplantation experiments were perfomed by a researcher blinded to the transplanted organoid identity

Reporting for specific materials, systems and methods

We require information from authors about some types of materials, experimental systems and methods used in many studies. Here, indicate whether each material, system or method listed is relevant to your study. If you are not sure if a list item applies to your research, read the appropriate section before selecting a response.

Materials & experimental systems

n/a	Involved in the study
☐	☒ Antibodies
☐	☒ Eukaryotic cell lines
☐	☐ Palaeontology and archaeology
☐	☒ Animals and other organisms
☐	☒ Clinical data
☐	☐ Dual use research of concern
☐	☐ Plants

Methods

n/a	Involved in the study
☐	☐ ChIP-seq
☐	☒ Flow cytometry
☐	☐ MRI-based neuroimaging

Antibodies

Antibodies used

Antibody/Source/Catalog/clone number/Dilution factor
- Antibodies used for immunofluorescent staining in this study:
Anti Human ACE Sigma-Aldrich HPA029298 1:200
Anti Human AGTR1 Bioss Antibodies BS-2097R 1:200
Anti Human ASS1 Abcam ab77590 1:100
Anti Human CD31 Novus Biologicals NBP2-80640 1:200
Anti Human CUBN Sigma-Aldrich SAB4301904 1:100
Anti Human E-Cadherin BD Transduction Laboratories™ 610182 1:350
Anti Human GATA3 BioTechne R&D AF2605 1:500
Anti Human HNF4a Abcam ab41898 1:200
Anti Human Kim1 R&D Systems AF1750 1:200
Anti Human KRT8/18 Abcam ab194130 1:1000
Anti Human LIM1/LHX1 Abcam ab229474 1:250
Anti Human LRP2/Megalin BioTechne R&D MAB9578-100 1:100
LTL (Biotinylated) Vector Laboratories B-1325-2 1:500
Anti Human Nephrin/NPHS1 R&D Systems AF4269 1:300
Anti Human Podocalyxin BioTechne R&D MAB1658 1:50
Anti Human Podocin/NPHS2 Proteintech 20384-1-AP 1:100
Anti Human SLC22A2 Abcam ab170871 1:100
Anti Human SLC34A1 ThermoFisher PA5-62358 1:100
Anti Human TFAP2B Cell Signaling Technology 2509S 1:200
Anti Human TROMA-1 Sigma-Aldrich MABT329 1:50
Anti Human VE-Cadherin Novus Biologicals NBP1-43347 1:200
Anti Human VEGF-A Abcam ab52917 1:250
Anti Human WT1 Santa Cruz sc-393498 1:20
Anti mosue VE Cadherin Novus Biologicals AF1002-SP 1:200
Anti mosue CD31 Cell Signaling Technology 77699T 1:200

- Seconadry antibodies used in this study
Donkey Anti-Rabbit IgG H&L (Alexa Fluor 488) Abcam ab150073 1:400
Donkey Anti-Mouse IgG H&L (Alexa Fluor 488) Abcam ab150105 1:400
Donkey Anti-Goat IgG H&L (Alexa Fluor 647) Abcam ab150131 1:400
Donkey Anti-Rat IgG H&L (Alexa Fluor 647) Abcam ab150155 1:400
Donkey Anti-Rabbit IgG H&L (Alexa Fluor 568) Abcam ab175470 1:400
Donkey F(ab')2 Anti-Mouse IgG H&L (Alexa Fluor 568) Abcam ab175699 1:400
Streptavidin, Alexa Fluor™ 405 conjugate Invitrogen S32351 1:400

- Antibodies used for Flow Cytometry in this study
Anti human ACE conjugated to APC Miltenyi Biotech, clone: REA522 catalog no. 130-108-014 1:11
human IgG1 Isotype control Miltenyi Biotech 130-113-446 1:50
Anti human ATIR conjugated to APC Novus Biologicals, Clone: 1010103, catalog no. FAB10244A 1:11
IgG2b kappa Isotype Control eBioscience™ 17-4031-82 1:100
7-AAD eBioscience™ 00-6993-50 1:11

Validation

All antibodies used in this study have been validated by other and our research group in previous studies and are commonly used in the field. Each antibody was selected based on the stated species reactivity by the provider website, its validation for use for the desired method (i.e. P-IHC, Flow cytometry) and the quality of citations provided. Validation for many of the antibodies used in the study can also be found in the ReBuilding a Kidney (RBK) "Antibody Validation" section (URL: https://www.rebuildingakidney.org/).

Eukaryotic cell lines

Policy information about cell lines and Sex and Gender in Research

Cell line source(s)	Human induced pluripotent cell lines:<br>- NHSK: Derived from Normal Human Dermal Keratinocyte of a female (XX) healthy individual, generated at Cincinnati Children's Hospital Pluripotent Stem Cell Facility<br>Generation of iPSC lines with CRISPR/CAS ACE or AGTR1 gene deletion (ACE-/-, AGTR1-/-) and their respective isogenic controls (unmodified) was perfomed with NHSK iPSC as the parental line.<br>- P-ACE: Derived from urine epithelial cells collected from a 9-year-old female donor AR-RTD patient harboring biallelic pathogenic variant in the ACE gene (c.2570G>A) leading to Arginine (R) to Histidine (H) substitution. CRISPR/Cas9-mediated gene editing was used to generate an isogenic corrected iPSC (C-ACE) with P-ACE iPSC as the parental line.
Authentication	Authentication using Short Tandem Repeat (STR) analysis was performed for all parental (NHSK, P-ACE) and CRISPR-modifed (ACE-/-, AGTR1-/- and C-ACE) hiPSC and all lines used in this study passed this analysis successfully.<br>Successful differentiation into kidney organoids is an additional authentication for the hiPSC used in this study.
Mycoplasma contamination	All cell lines were periodically tested for mycoplama contamination via PCR and only verified mycoplasma-negative lines were used for all the experiments in this manuscript
Commonly misidentified lines (See ICLAC register)	Only validated hiPSC lines were included in the study

Palaeontology and Archaeology

Specimen provenance	*Provide provenance information for specimens and describe permits that were obtained for the work (including the name of the issuing authority, the date of issue, and any identifying information). Permits should encompass collection and, where applicable, export.*
Specimen deposition	*Indicate where the specimens have been deposited to permit free access by other researchers.*
Dating methods	*If new dates are provided, describe how they were obtained (e.g. collection, storage, sample pretreatment and measurement), where they were obtained (i.e. lab name), the calibration program and the protocol for quality assurance OR state that no new dates are provided.*

☐ Tick this box to confirm that the raw and calibrated dates are available in the paper or in Supplementary Information.

Ethics oversight	*Identify the organization(s) that approved or provided guidance on the study protocol, OR state that no ethical approval or guidance was required and explain why not.*

Note that full information on the approval of the study protocol must also be provided in the manuscript.

Animals and other research organisms

Policy information about studies involving animals; ARRIVE guidelines recommended for reporting animal research, and Sex and Gender in Research

Laboratory animals	In this study 8 week old, NOD.Cg-Prkdcscid Il2rgtm1Wjl/SzJ ('NOG') mice, strain #0055578, were used as graft recipients
Wild animals	This study did not involve wild animals
Reporting on sex	Both male and female mice were used as recipients. No difference in recipient sex affected engraftment success or organoid differentiation.
Field-collected samples	The study did not involve samples collcted from the field
Ethics oversight	Our experimental protocols (IACUC no. 2021-0060) was approved by the Animal Studies Committee of Cincinnati Children's Hopital Medical Center. Mice were maintained at the Cincinnati Children's Hospital Medical Center animal facility following animal care guidelines. Animals are housed in sterile (autoclaved) cages on individually ventilated racks. The feed and bedding is autoclaved. Water is UV sterilized and delivered from automatic water system on the racks. All mice are housed in Specific pathogen free barrier facility. Animals are monitored daily for activity/health and any deviations are addressed by technicians under the direction of veterinarians. CCHMC vivarium is AAALAC accredited and maintains top quality animal care. Dark/Light cycle: Our animal facility is on 14 hour light and 10 hour dark cycle through out the year. Light cycle is programmed and managed by building management software. Temperature for all animal rooms is set to 72F. The temperature is also monitored by building management software system with alerts to veterinary staff for any deviations beyond the setpoint. Relative Humidity of the animal holding rooms is maintained within the acceptable rage of 30 to 80% as suggested by the guide for the care and use of laboratory animals. Our experimental protocols (IACUC no. 2021-0060) were approved by the Institutional Animal Care and Use Committee of CCHMC.

Note that full information on the approval of the study protocol must also be provided in the manuscript.

Clinical data

Policy information about clinical studies

All manuscripts should comply with the ICMJE guidelines for publication of clinical research and a completed CONSORT checklist must be included with all submissions.

Clinical trial registration	*Provide the trial registration number from ClinicalTrials.gov or an equivalent agency.*
Study protocol	*Note where the full trial protocol can be accessed OR if not available, explain why.*
Data collection	*Describe the settings and locales of data collection, noting the time periods of recruitment and data collection.*
Outcomes	*Describe how you pre-defined primary and secondary outcome measures and how you assessed these measures.*

Dual use research of concern

Policy information about dual use research of concern

Hazards

Could the accidental, deliberate or reckless misuse of agents or technologies generated in the work, or the application of information presented in the manuscript, pose a threat to:

No	Yes	
☒	☐	Public health
☒	☐	National security
☒	☐	Crops and/or livestock
☒	☐	Ecosystems
☒	☐	Any other significant area

Experiments of concern

Does the work involve any of these experiments of concern:

No	Yes	
☒	☐	Demonstrate how to render a vaccine ineffective
☒	☐	Confer resistance to therapeutically useful antibiotics or antiviral agents
☒	☐	Enhance the virulence of a pathogen or render a nonpathogen virulent
☒	☐	Increase transmissibility of a pathogen
☒	☐	Alter the host range of a pathogen
☒	☐	Enable evasion of diagnostic/detection modalities
☒	☐	Enable the weaponization of a biological agent or toxin
☒	☐	Any other potentially harmful combination of experiments and agents

Plants

Seed stocks	*Report on the source of all seed stocks or other plant material used. If applicable, state the seed stock centre and catalogue number. If plant specimens were collected from the field, describe the collection location, date and sampling procedures.*
Novel plant genotypes	*Describe the methods by which all novel plant genotypes were produced. This includes those generated by transgenic approaches, gene editing, chemical/radiation-based mutagenesis and hybridization. For transgenic lines, describe the transformation method, the number of independent lines analyzed and the generation upon which experiments were performed. For gene-edited lines, describe the editor used, the endogenous sequence targeted for editing, the targeting guide RNA sequence (if applicable) and how the editor was applied.*
Authentication	*Describe any authentication procedures for each seed stock used or novel genotype generated. Describe any experiments used to assess the effect of a mutation and, where applicable, how potential secondary effects (e.g. second site T-DNA insertions, mosiacism, off-target gene editing) were examined.*

ChIP-seq

Data deposition

☐ Confirm that both raw and final processed data have been deposited in a public database such as GEO.

☐ Confirm that you have deposited or provided access to graph files (e.g. BED files) for the called peaks.

Data access links<br>*May remain private before publication.*	*For "Initial submission" or "Revised version" documents, provide reviewer access links. For your "Final submission" document, provide a link to the deposited data.*
Files in database submission	*Provide a list of all files available in the database submission.*
Genome browser session<br>(e.g. UCSC)	*Provide a link to an anonymized genome browser session for "Initial submission" and "Revised version" documents only, to enable peer review. Write "no longer applicable" for "Final submission" documents.*

Methodology

Replicates	*Describe the experimental replicates, specifying number, type and replicate agreement.*
Sequencing depth	*Describe the sequencing depth for each experiment, providing the total number of reads, uniquely mapped reads, length of reads and whether they were paired- or single-end.*
Antibodies	*Describe the antibodies used for the ChIP-seq experiments; as applicable, provide supplier name, catalog number, clone name, and lot number.*
Peak calling parameters	*Specify the command line program and parameters used for read mapping and peak calling, including the ChIP, control and index files used.*
Data quality	*Describe the methods used to ensure data quality in full detail, including how many peaks are at FDR 5% and above 5-fold enrichment.*
Software	*Describe the software used to collect and analyze the ChIP-seq data. For custom code that has been deposited into a community repository, provide accession details.*

Flow Cytometry

Plots

Confirm that:

☒ The axis labels state the marker and fluorochrome used (e.g. CD4-FITC).

☒ The axis scales are clearly visible. Include numbers along axes only for bottom left plot of group (a 'group' is an analysis of identical markers).

☐ All plots are contour plots with outliers or pseudocolor plots.

☒ A numerical value for number of cells or percentage (with statistics) is provided.

Methodology

Sample preparation	Single cell suspension from ACE-/-, AGTR1-/-, P-ACE iPSC-derived organoids at day 24 were collected from transwells (6-8 per cell line and condition), transferred into a 15ml tube containing 1ml of TrypLE (Invitrogen), mechanically disrupted via pipetting and incubated for 5min at 37Ċ. Next, organoids were washed by adding 6ml of PBS to each tube, centrifuged at 1200rpm for 4 min, and the supernatant was aspirated. 200 µL of collagenase IV (STEMCELL Technologies) was added, and samples were incubated for 10 min at 37Ċ. Following additional pipetting, 6 mL of PBS was added to each tube and samples were centrifuged again. Supernatant was removed and each single cell suspension was resuspended with FACS buffer (PBS containing 0.2% BSA; BD Biosciences) and distributed into 1.5ml Eppendorf tubes at 1×105 to 5×105 per tube, centrifugated and resuspended in 100 µl of blocking buffer (FACS buffer containing Fc block at 1:50 ratio) (BD Biosciences)). Following incubation for 10min, primary conjugated antibodies, isotype controls and dead cell staining (7-AAD, eBiosystems) were added to each tube and incubated in the dark for 45 min on ice. For ACE-/- and P-ACE iPSC derived organoids and respective IC, an anti-human ACE:APC (Miltenyi Biotec) was used and for AGTR1-/- and IC organoids an anti-human AT1R:APC (Novus Biologicals) was used. Next, vials were washed x2 with FACS buffer and resuspended in 250ul, prior to analysis on the BD FACS Canto II.
Instrument	BD/FACSCanto II
Software	The flow cytometry results were analyzed using FlowJo™ v10.8 Software (BD Life Sciences).
Cell population abundance	No FACS sorting experiments were perfomred in this study.
Gating strategy	The gating strategy included identification of live cells based on forward and side scatter properties, followed by exclusion of dead cells based on staining with based on staining with 7-aminoactinomycin D (7AAD) and forward scatter pulse width. After

defining the parent gate, subsequent gating was performed to exclude non-specific background fluorescence and to identify and select the target cell population with greater specificity using antibody-specific isotype controls. The data obtained from this gate was used for downstream analysis.
The target cell population was subsequently defined based on fluorescence intensity of either anti-human ACE or AT1R antibody conjugated to APC fluorochrome.
All flow cytometry data were analyzed using FlowJo software, and the percentage of cells in each gate was recorded for further analysis.

☐ Tick this box to confirm that a figure exemplifying the gating strategy is provided in the Supplementary Information.

Magnetic resonance imaging

Experimental design

Design type — *Indicate task or resting state; event-related or block design.*

Design specifications — *Specify the number of blocks, trials or experimental units per session and/or subject, and specify the length of each trial or block (if trials are blocked) and interval between trials.*

Behavioral performance measures — *State number and/or type of variables recorded (e.g. correct button press, response time) and what statistics were used to establish that the subjects were performing the task as expected (e.g. mean, range, and/or standard deviation across subjects).*

Acquisition

Imaging type(s) — *Specify: functional, structural, diffusion, perfusion.*

Field strength — *Specify in Tesla*

Sequence & imaging parameters — *Specify the pulse sequence type (gradient echo, spin echo, etc.), imaging type (EPI, spiral, etc.), field of view, matrix size, slice thickness, orientation and TE/TR/flip angle.*

Area of acquisition — *State whether a whole brain scan was used OR define the area of acquisition, describing how the region was determined.*

Diffusion MRI ☐ Used ☐ Not used

Preprocessing

Preprocessing software — *Provide detail on software version and revision number and on specific parameters (model/functions, brain extraction, segmentation, smoothing kernel size, etc.).*

Normalization — *If data were normalized/standardized, describe the approach(es): specify linear or non-linear and define image types used for transformation OR indicate that data were not normalized and explain rationale for lack of normalization.*

Normalization template — *Describe the template used for normalization/transformation, specifying subject space or group standardized space (e.g. original Talairach, MNI305, ICBM152) OR indicate that the data were not normalized.*

Noise and artifact removal — *Describe your procedure(s) for artifact and structured noise removal, specifying motion parameters, tissue signals and physiological signals (heart rate, respiration).*

Volume censoring — *Define your software and/or method and criteria for volume censoring, and state the extent of such censoring.*

Statistical modeling & inference

Model type and settings — *Specify type (mass univariate, multivariate, RSA, predictive, etc.) and describe essential details of the model at the first and second levels (e.g. fixed, random or mixed effects; drift or auto-correlation).*

Effect(s) tested — *Define precise effect in terms of the task or stimulus conditions instead of psychological concepts and indicate whether ANOVA or factorial designs were used.*

Specify type of analysis: ☐ Whole brain ☐ ROI-based ☐ Both

Statistic type for inference — *Specify voxel-wise or cluster-wise and report all relevant parameters for cluster-wise methods.*

(See Eklund et al. 2016)

Correction — *Describe the type of correction and how it is obtained for multiple comparisons (e.g. FWE, FDR, permutation or Monte Carlo).*

Models & analysis

n/a	Involved in the study
☐	☐ Functional and/or effective connectivity
☐	☐ Graph analysis
☐	☐ Multivariate modeling or predictive analysis

Functional and/or effective connectivity	*Report the measures of dependence used and the model details (e.g. Pearson correlation, partial correlation, mutual information).*
Graph analysis	*Report the dependent variable and connectivity measure, specifying weighted graph or binarized graph, subject- or group-level, and the global and/or node summaries used (e.g. clustering coefficient, efficiency, etc.).*
Multivariate modeling and predictive analysis	*Specify independent variables, features extraction and dimension reduction, model, training and evaluation metrics.*
